# Three chemotypes of thyme (*Thymus vulgaris* L.) essential oil and their main compounds affect differently the IL-6 and TNFα cytokine secretions of BV-2 microglia by modulating the NF-κB and C/EBPβ signalling pathways

**DOI:** 10.1186/s12906-021-03319-w

**Published:** 2021-05-22

**Authors:** Györgyi Horváth, Adrienn Horváth, Gréta Reichert, Andrea Böszörményi, Katalin Sipos, Edina Pandur

**Affiliations:** 1 Department of Pharmacognosy, Faculty of Pharmacy, University of Pécs, H-7624, Rókus u. 2., Pécs, Hungary; 2 Institute of Pharmacognosy, Faculty of Pharmacy, Semmelweis University, H-1085 Üllői út 26, Budapest, Hungary; 3 Department of Pharmaceutical Biology, Faculty of Pharmacy, University of Pécs, H-7624, Rókus u. 2, Pécs, Hungary

**Keywords:** Essential oil, Inflammation, Microglia, Cytokine, NF-κB, C/EBPβ

## Abstract

**Background:**

The essential oils possess both antimicrobial and anti-inflammatory effects, therefore they can provide an effective treatment against infections. Essential oils are widely used as supportive ingredients in many diseases, especially in the acute and chronic diseases of the respiratory tract. Neuroinflammation is responsible for several diseases of the central nervous system. Some plant-derived bioactive molecules have been shown to have role in attenuating neuroinflammation by regulating microglia, the immune cells of the CNS.

**Methods:**

In this study, the anti-inflammatory effect of three chemotypes of thyme essential oil and their main compounds (geraniol, thujanol and linalool) were examined on lipopolysaccharide-induced BV-2 microglia. Three different experimental setups were used, LPS pretreatment, essential oil pretreatment and co-treatments of LPS and essential oils in order to determine whether essential oils are able to prevent inflammation and can decrease it. The concentrations of the secreted tumour necrosis factor α (TNFα) and interleukin-6 (IL-6) proinflammatory cytokines were measured and we analysed by Western blot the activity of the cell signalling pathways, NF-κB and CCAAT-enhancer binding protein β (C/EBPβ) regulating TNFα and IL-6 proinflammatory cytokine expressions in BV-2 cells.

**Results:**

Our results showed definite alterations in the effects of essential oil chemotypes and their main compounds at the different experimental setups. Considering the changes of IL-6 and TNFα secretions the best reduction of inflammatory cytokines could be reached by the pretreatment with the essential oils. In addition, the main compounds exerted better effects than essential oil chemotypes in case of LPS pretreatment. At the essential oil pretreatment experiment, the effect of linalool and geraniol was outstanding but there was no major difference between the actions of chemotypes and standards. Main compounds could be seen to have large inhibitory effects on certain cell signalling components related to the activation of the expression of proinflammatory cytokines.

**Conclusion:**

Thyme essential oils are good candidates to use in prevention of neuroinflammation and related neurodegeneration, but the exact ratio of the components has to be selected carefully.

**Supplementary Information:**

The online version contains supplementary material available at 10.1186/s12906-021-03319-w.

## Background

Today there is an increasing demand for the use of natural ingredients and their derivatives in the treatment of different health problems. Among them essential oils enjoy popularity, which are commonly used nowadays in cosmetics, health care, traditional medicine and food industry. Because of their antimicrobial activities, the application of the essential oils is widespread. They have a complex mode of action due to their multiple composition. The composition of essential oils is variable and the physiological action and organoleptic characteristic is dominated by the major constituent defined by chemotype [1, 2].

The volatile essential oils can easily reach the upper and lower parts of the respiratory tract via inhalation. They possess both antimicrobial and anti-inflammatory effects, therefore they can provide an effective treatment against infections [3–6]. After infection several molecular and cellular events play role in stimulating initial acute inflammation, which leads to the accumulation of leukocytes and plasma proteins induced by cytokines derived from protector cells like dendritic, mast, endothelial cells and macrophages [7, 8].

Microglia, the immune cells of the central nervous system (CNS) are activated at inflammation process and produce inflammatory cytokines, which may impair the function of nerve cells causing cell death [9]. Therefore, the role of the anti-inflammatory extracts and their components obtained from plants are highly important. Furthermore, neuroinflammation is responsible for several CNS diseases (e.g. neurodegenerative disorders, depression, sleep disorders, and stroke) [10–12]. To prevent neuroinflammation there is possibility to cure these disorders. Some plant-derived bioactive molecules have been shown to have role in attenuating neuroinflammation [11, 13–15]. Recent evidence has proven that the essential oils can transfer through the nasal mucosa during inhalation, can enter the blood circulation and pass through the blood-brain barrier [16, 17].

Because of the great number of constituents, essential oils seem to have several potential cellular targets [18]. Pérez et al. [6] summarized the anti-inflammatory properties of some essential oils and their proposed or studied mechanism of action. These mode of actions include various processes, e.g. modulation of lipoxygenase enzymatic activity, nitric oxide (NO) inhibition, inhibition of secretion of proinflammatory cytokines like tumour necrosis factor α (TNFα) and interleukin-1β (IL-1β), and inhibition of NF-κB activation.

Essential oil of thyme (*Thymus vulgaris* L.) is utilized as complementary therapy of acute and chronic diseases of the respiratory tract [19, 20]. The diverse biological activities of thyme oil are related to its main phenolic compounds, thymol and carvacrol [21, 22]. The anti-inflammatory effect of thyme oil and some of its main components has been widely studied and proved using mice models [23, 24] and cells like THP-1 (human acute monocytic leukaemia cell line) [25], J774A.1 (murine macrophage cell line) [26, 27], human polymorphonuclear neutrophils [28] and RAW 264.7 (murine macrophage cell line) [29].

In our previous studies we have demonstrated the antibacterial activity of thyme essential oil against some respiratory pathogens [30, 31]. Due to its antimicrobial and anti-inflammatory potency, it may offer an effective treatment in neuroinflammation. However, its role in the mechanism of neuroinflammation is not fully understood [11].

The aim of this study was to examine the anti-inflammatory effect of three chemotypes of thyme essential oil and their main compounds on lipopolysaccharide (LPS)-induced BV-2 microglia. Furthermore, this study is the first in which the anti-inflammatory effect of geraniol and thujanol chemotypes of thyme oil (*Thymus vulgaris* L.) and their main compounds (geraniol and thujanol) was examined on BV-2 microglia.

Our results unravelled that thyme oil chemotypes and their main compounds possess anti-inflammatory effect on LPS-induced microglia via modulating the activation of NF-κB and C/EBPβ signalling pathways and decreasing the secretion of IL-6 and TNFα proinflammatory cytokines. It was demonstrated that chemotypes and the main compounds exerted different inhibitory effects on the examined signalling proteins. Based on our results we suppose that development of an essential oil product containing the major compounds of thyme essential oil in a proper ratio would be successful as complementary neurotherapeutics against neuroinflammation.

## Methods

### Essential oils

Three chemotypes of *Thymus vulgaris* essential oil, linalool (Lot number: OF16244), geraniol (Lot number: OF7289) and thujanol (Lot number: OF19102) were purchased from Panarom (Panarom Naturkozmetika Kft., Budapest, Hungary). Linalool, geraniol and thujanol essential oil standards were purchased from Sigma-Aldrich (Sigma-Aldrich Kft., Budapest, Hungary). Stock solutions of the chemotypes were produced by adding 100 μL of pure dimethyl sulfoxide (DMSO, Sigma-Aldrich Kft., Budapest, Hungary) to 900 μL of essential oil, therefore the stock solution contained 90% of essential oil and 10% of DMSO. The emulsions were mixed by vortexing then were diluted with phosphate buffered saline (PBS, Lonza Ltd., Basel, Switzerland) 200-fold, 500-fold and 1000-fold. Stock solutions of linalool and geraniol standards were prepared the same way as the chemotypes. Stock solutions of thujanol standard was prepared by solving 4 mg of thujanol in 1 mL of DMSO. Dilutions of the standard stock solutions were carried out the same way as in case of the essential oil chemotypes. For control experiments 10% DMSO stock solution was prepared in PBS and was diluted the same way as the essential oils, 200-fold, 500-fold and 1000-fold. The final concentrations of DMSO used in the experiments were 0.05, 0.02 and 0.01% according to the dilutions.

### GC-MS analysis

The chemical composition of the thyme oil chemotypes was analysed by gas chromatography-mass spectrometry (GC-MS). A 1 μL of each essential oil sample was diluted in ethanol (10 μL/mL) then it was injected in split mode. The temperature of the injector was 250 °C, the split ratio was 1:10. The analyses were carried out with an Agilent 6890 N/5973 N GC-MSD (Santa Clara, CA, USA) system equipped with a Supelco (Sigma-Aldrich Kft., Budapest, Hungary) SLB-5MS capillary column (30 m × 250 μm × 0.25 μm). The GC oven temperature increased from 60 °C (3 min isothermal) to 250 °C at 8 °C /min (1 min isothermal). The carrier gas was high purity helium (6.0; at 1.0 mL/min (37 cm/s)) in a constant flow mode. The mass selective detector (quadrupole mass analyser) was operated in electron ionization mode at 70 eV in a full scan mode (41–500 amu at 3.2 scan/s). The data were analysed using MSD ChemStation D.02.00.275 software (Agilent Technologies, Santa Clara, CA). The identification of the compounds was carried out by comparing retention times and recorded spectra with the data of authentic standards involving the NIST 2.0 library. The calculation of the percentage was carried out by area normalization [30].

### Cell culture and treatments

BV-2 murine microglial cells (kind gift from Prof. László Tretter and his research group) were maintained in Dulbecco’s Modified Eagle Medium (DMEM; Lonza Ltd., Basel, Switzerland) supplemented with 10% fetal bovine serum (FBS, EuroClone S.p.A, Pero, Italy) and 1% penicillin/streptomycin (Lonza Ltd., Basel, Switzerland). The cells were cultured on poly-L-ornithine (Sigma-Aldrich Kft., Budapest, Hungary) coated dishes (Sarstedt Kft., Budapest, Hungary). BV-2 cells were seeded into 6-well plates and were cultured for 24 h before the treatments. The cells were treated with 200-fold diluted essential oil chemotypes and standards to determine their effects on cytokine production. Inflammatory condition was generated by LPS treatment (1 μg/mL, *Escherichia coli* O55:B5, Sigma-Aldrich Kft., Budapest, Hungary). Anti-inflammatory effects of essential oils were determined in three different experiments: LPS pretreatment for 24 h then essential oil treatment for 24 h; essential oil pretreatment for 24 h then LPS treatment for 24 h; and co-treatment with LPS and essential oils for 24 h. DMSO treated cells were used as controls. The final concentrations of DMSO used in the experiments were 0.05, 0.02 and 0.01% according to the dilutions. Each experiments were repeated at least three times. All experiments were carried out in a humidified atmosphere containing 5% CO_2_ at 37 °C.

### Cell viability assay

BV-2 cells were plated onto 96-well plates using 5 × 10^3^ cells/well. Cells were treated with essential oils and standards in 200-fold, 500-fold and 1000-fold dilutions for 6 h and 24 h. Viability of the BV-2 cells were measured using Cell Counting Kit-8 (CCK-8) cell viability assay (Sigma-Aldrich Kft., Budapest, Hungary) after the treatments. DMSO treated cells were used as controls of the essential oil treated cells, while the effect of DMSO on cell viability was determined by using untreated cells as controls. After each treatment 10 μL of WST-8 reagent was added to each well, then the plates were incubated for 1 h at 37 °C and 5% CO_2_. After incubation, 10 μL of 1% sodium-dodecyl sulphate (SDS, Molar Chemicals Kft., Halásztelek, Hungary) was added to each well to stop the reaction. The absorbance of the samples was measured at 450 nm using MultiSkan GO microplate spectrophotometer (Thermo Fisher Scientific Inc., Waltham, MA). Viability was expressed as percentile of the total cell number of the appropriate control.

### Real-time PCR

BV-2 cells were treated the same way as described earlier, in 6-well culture dishes (3 × 10^5^ cells/well). After the treatments, BV-2 cells were washed with PBS and then were collected after trypsinization. Total RNA was isolated from each sample using Quick RNA mini kit (Zymo Research, Irvine, CA). Complementary DNA was synthesised from 200 ng of total RNA using High capacity cDNA Reverse Transcription Kit (Applied Biosystems, Thermo Fisher Scientific Inc., Waltham, MA) according to the manufacturer’s protocol. Determination of gene expressions was performed in a CFX96 Real-time System (Bio-Rad Inc., Hercules, CA) using iTaq™ Universal SYBR® Green Supermix (Bio-Rad Inc., Hercules, CA) in a 20 μL of total reaction volume. Melting curves were generated after each quantitative PCR run to ensure that a single specific product was amplified. Relative quantification was calculated by the Livak (∆∆Ct) method using the Bio-Rad CFX Maestro software (Bio-Rad Inc., Hercules, CA). The expression level of the gene of interest was compared with the level of β-actin in each sample. These relative expression rates were then compared between the treated and the untreated samples. The relative expression of the controls was regarded as 1 [31]. The mRNA expression of the treated cells were compared to the controls. The primer sequences used in this study are described in Table 1.

**Table 1:** Real-time PCR gene primer list

Primer	Sequence 5′ → 3’
IL-6 forward	CTCTGCAAGAGACTTCCATCCA
IL-6 reverse	GACAGGTCTGTTGGGAGTGG
TNFα forward	GATCGGTCCCCAAAGGGATG
TNFα reverse	CCACTTGGTGGTTTGTGAGTG
β-actin forward	CTGTCGAGTCGCGTCCA
β-actin reverse	TCATCCATGGCGAACTGGTG

### Enzyme-Linked Immunosorbent Assay (ELISA) Measurements

After each treatment of the cells, culture media of the control and treated cells were collected and stored at − 80 °C until the measurements. Secreted IL-6 and TNFα concentrations of the culture media were determined with mouse IL-6 and mouse TNFα ELISA Kits (Thermo Fisher Scientific Inc., Waltham, MA) according to the instructions of the manufacturer [32].

### Immunoblotting

BV-2 cells were seeded onto 6-well culture dishes (3 × 10^5^ cells/well) and were treated after a 24 h incubation period. BV-2 cells were fractionated immediately after collection using Subcellular Protein Fractionation Kit for Cultured Cells (Thermo Fisher Scientific Inc., Waltham, MA) according to the manufacturer’s protocol. Protein contents of each protein fraction were measured with DC Protein Assay Kit (Bio-Rad Inc., Hercules, CA). The same amount of protein (15 μg) from each sample was loaded onto 10% or 12% SDS-polyacrylamide gels. After the electrophoresis the protein content of the gels were transferred by electro- blotting to nitrocellulose membranes (Pall AG, Basel, Switzerland). The membranes were blocked with 5% non-fat dry milk in TBST (Tris buffer saline, 0.1% Tween-20) for 1 h at room temperature [33]. After the blocking step, the membranes were probed with the following polyclonal rabbit antibodies for overnight at 4 °C according to the manufacturer’s protocols: anti-NF-κB/p50 IgG (1:1000, Sigma-Aldrich Kft., Budapest, Hungary), anti-NF-κB/p65 IgG (1:2000, Cell Signaling Technology Europe, Leiden, The Netherlands) and anti-phospho-C/EBPβ IgG (1:1000, Thermo Fisher Scientific Inc., Waltham, MA). β-actin (1:2000; Sigma-Aldrich Kft., Budapest, Hungary) was used as housekeeping control in all Western blot experiments. Goat anti-rabbit HRP-conjugate was used as secondary antibody (1:3000; Bio-Rad Inc., Hercules, CA). Protein detection was carried out with WesternBright ECL chemiluminescent substrate (Advansta Inc., San Jose, CA). Optical densities of Western blots were determined using ImageJ software [34], and were expressed as percentage of target protein/β-actin abundance.

### Statistical analysis

The data presented are representative of at least three independent experiments. For all data, *n* corresponds to the number of independent experiments. Real-time PCR and cell viability assays and ELISA measurements were carried out in triplicate in each independent experiments. Statistical analysis was performed using SPSS software (IBM Corporation, Armonk, NY, USA). Statistical significance was determined by Kruskal-Wallis one-way ANOVA non-parametric test using pairwise comparisons [33]. Data are shown as mean ± standard deviation (SD). The difference between means was determined at 95% confidence intervals. Statistical significance was set at *p* value < 0.05*.*

## Results

### Effects of essential oils on cell viability of BV-2 cells

**Fig. 1:**
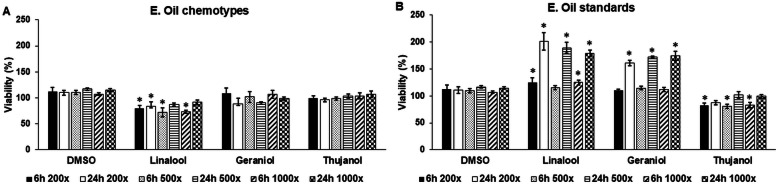
Cell viability determinations of the treated BV-2 cells. Viability of the BV-2 cells was measured using CCK-8 cell viability assay after the 6 h and 24 h long treatments with DMSO, chemotypes or standards. Viability is expressed as percentile of the total cell number of the control cells. **a** Cell viability measurements of BV-2 cells treated with DMSO or essential oil chemotypes. **b** Cell viability measurements of BV-2 cells treated with DMSO or essential oil standards. The bars represent mean values and error bars represent standard deviation (SD) for four independent determinations (*n* = 4). Cell viability assays were carried out in triplicate in each independent experiments. Asterisks indicate *p* < 0.05 compared to the DMSO treated cells used as controls

According to the GC-MS, geraniol (54.9%), thujanol (33.9%) and linalool (69.2%) were detected as main compounds in the three thyme oil chemotypes. Treatments with essential oils might be harmful to the cells [35–37], therefore their effects on cell viability in different dilutions (200-fold, 500-fold and 1000-fold) at 6 h and 24 h long treatments were examined DMSO was used as a carrier in the stock solutions, therefore, the effect of DMSO on the cells was also determined. DMSO did not affect significantly the cell viability (Fig. 1a,b). The three different chemotypes of the thyme essential oil acted similarly on the BV-2 cells, they did not caused significant changes on viability. The only exception was linalool that decreased significantly the viability at 6 h in each dilutions (Fig. 1b). On the contrary, essential oil standards caused remarkable alterations in cell viability. Both linalool and geraniol increased the viability of BV-2 cells at 6 h and 24 h, in the latter time point the elevation was significant (Fig. 1b). Meanwhile, thujanol standard decreased the viability at 6 h, but increased it at 24 h (Fig. 1b). Based on the results it seems that neither the standards nor the chemotypes were not toxic for the cells at 24 h. Therefore we chose the 200-fold dilution of essential oil chemotypes and standards for further studies. Percentage (%) and relative concentrations (μg/mL) of the main compounds of different chemotype thyme oils can be seen in Table 2. The compositions of the examined essential oil chemotypes can be seen in Table 3.

**Table 2:** Percentage (%) and relative concentrations (μg/mL) of the main compounds of different chemotype thyme oils used in the experiments and the dilution of the main oil compounds without cytotoxic effect

Compound	LRI	Percentage of compound in the oil^a^	Relative concentration of compound in the experiments^b^ (μg/mL)	Dilution of compound without cytotoxic effect
Geraniol	1253	54.9	2.1	200
Linalool	1104	69.2	2.7	200
Thujanol	1154	33.9	0.6	200

^a^LRI - linear retention index on SLB-5MS column

^b^The relative concentrations were chosen based on the calculation as if the cells were treated with 200 μL essential oil

**Table 3:** Percentage (%) and relative concentrations (μg/mL) of the compounds of different chemotype thyme oils

		Percentage of compound in the thyme chemotypes^a^			Relative concentration of compound in the experiments^b^ (μg/mL)		
Compound	LRI	Geraniol	Thujanol	Linalool	Geraniol	Thujanol	Linalool
α-Thujene	930	ND	0.4	0.6	ND	0.018	0.028
α-Pinene	939	0.1	1.2	1.4	0.004	0.051	0.061
Camphene	951	0.3	0.2	0.4	0.012	0.0084	0.017
1-Octen-3-ol	978	0.1	ND	ND	0.004	ND	ND
β-Myrcene	979	0.1	0.7	1.0	0.004	0.028	0.04
β-Pinene	992	ND	0.4	0.1	ND	0.017	0.004
α-Phellandrene	1003	ND	1.2	0.1	ND	0.051	0.004
α-Terpinene	1017	ND	1.4	0.1	ND	0.059	0.004
p-Cymene	1026	0.2	2.1	4.2	0.009	0.090	0.18
Limonene	1029	0.1	1.8	1.3	0.004	0.076	0.055
1,8-Cineole	1046	0.2	0.4	0.9	0.009	0.018	0.041
γ-Terpinene	1060	0.1	2.9	1.3	0.004	0.123	0.055
Terpinolene	1093	ND	0.7	1.2	ND	0.031	0.052
Linalool	1104	3.0	8.4	69.2	0.128	0.361	2.736
Myrcenol	1123	0.1	3.2	ND	0.004	0.136	ND
cis-β-Terpineol	1144	0.2	ND	1.5	0.009	ND	0.007
Verbenol	1145	ND	0.2	0.1	ND	0.001	0.001
Camphor	1146	0.1	0.1	1.2	0.005	0.005	0.059
trans-Thujanol	1154	ND	33.9	ND	ND	0.616	ND
cis-Thujanol	1159	ND	6.5	ND	ND	0.033	ND
trans-β-Terpineol	1163	ND	0.8	0.1	ND	0.037	0.005
Borneol	1169	1.5	0.4	2.3	0.008	0.002	0.012
Terpinen-4-ol	1177	0.6	11.9	8.3	0.028	0.555	0.373
α-Terpineol	1190	ND	4.3	1.8	ND	0.204	0.084
trans- Piperitol	1208	ND	0.1	0.5	ND	0.005	0.024
Nerol	1230	1.9	4.2	0.1	0.084	0.185	0.004
Neral	1238	0.8	ND	ND	0.035	ND	ND
Geraniol	1253	54.9	1.9	ND	2.193	0.083	ND
Linalyl acetate	1257	0.3	2.9	0.6	0.013	0.130	0.027
Geranial	1267	1.3	ND	ND	0.058	ND	ND
Bornyl acetate	1289	0.2	ND	ND	0.010	ND	ND
Thymol	1297	1.4	0.7	0.9	0.067	0.034	0.043
Carvacrol	1299	0.7	ND	ND	0.034	ND	ND
Neryl acetate	1365	0.2	ND	ND	0.009	ND	ND
Geranyl acetate	1381	18.6	0.7	ND	0.851	0.032	ND
β-Caryophyllene	1417	5.7	4.0	0.6	0.3	0.180	0.027
α-Humulene	1452	0.1	ND	ND	0.004	ND	ND
Geranyl propionate	1486	2.2	ND	ND	0.099	ND	ND
Germacrene D	1486	ND	0.8	ND	ND	0.032	ND
Geranyl isobutyrate	1515	0.3	ND	ND	0.013	ND	ND
Elemol	1550	2.2	ND	ND	0.103	ND	ND
Caryophyllene oxide	1583	1.4	0.6	ND	0.067	0.029	ND
β-Eudesmol	1651	0.4	ND	ND	0.019	ND	ND
Total:		99.3	99.0	99.8			

^a^LRI - linear retention index on SLB-5MS column

^b^The relative concentrations were chosen based on the calculation as if the cells were treated with 200 μL essential oil

### Effects of essential oils on mRNA expression and secretion of IL-6 and TNFα

**Fig. 2:**
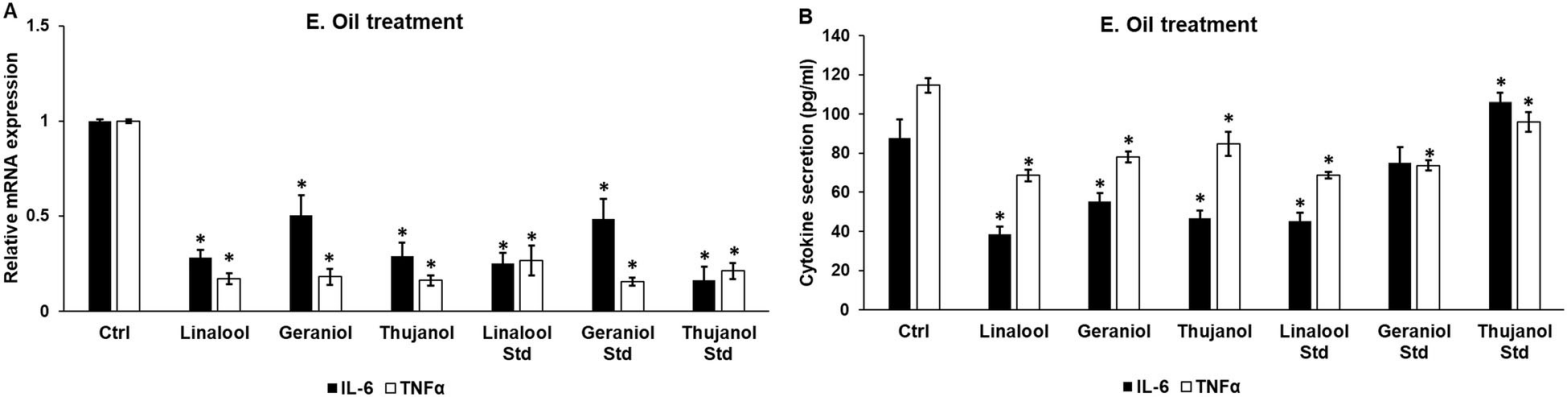
mRNA and protein levels of IL-6 and TNFα after essential oil treatments of BV-2 cells. BV-2 cells were treated with 200-fold diluted essential oil chemotypes and standards, and DMSO treatment was used as a control of the treated cells. Real-time PCR was performed with SYBR green protocol using IL-6 and TNFα specific primers. β-actin was used as housekeeping gene for the normalization and relative expression of controls was considered as 1. Proinflammatory cytokine secretions were determined using IL-6 or TNFα specific ELISA kits according to the manufacturer’s protocols. **a** Relative mRNA expressions of IL-6 and TNFα of the essential oil treated cells. **b** IL-6 and TNFα ELISA measurements from the supernatant of the essential oil treated BV-2 cells. The bars represent mean values and error bars represent standard deviation (SD) for three independent determinations (*n* = 3). Real-time PCR and ELISA measurements were carried out in triplicate in each independent experiments. Asterisks indicate *p* < 0.05 compared to the control

IL-6 and TNFα are proinflammatory cytokines, and their increased productions indicate the activation of the microglial cells. 24 h long treatments of BV-2 cells were carried out to determine their effects on the mRNA levels and protein secretions of IL-6 and TNFα cytokines. All of the examined essential oil chemotypes and their standards significantly decreased the mRNA expressions of IL-6 and TNFα compared to the DMSO treated control cells (Fig. 2a). All of the essential oil chemotypes decreased the IL-6 secretion (Fig. 2b). From the standards, only linalool was able to decrease significantly the IL-6 secretion, meanwhile thujanol standard significantly increased IL-6 protein level (Fig. 2b). ELISA measurements showed that the same three chemotypes, linalool, geraniol and thujanol decreased TNFα secretion suggesting that these chemotypes are good candidates for anti-inflammatory treatment against neuroinflammation. Linalool and geraniol standards were also successful in decreasing significantly the TNFα secretion. Thujanol standard treated cells showed also decreased TNFα level, but it could not be consider as a significant change (Fig. 2b).

### Essential oils alter mRNA expression and secretion of IL-6 and TNFα after lipopolysaccharide pretreatment

**Fig. 3:**
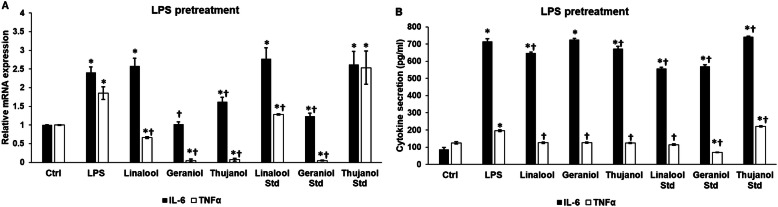
Effects of essential oils on mRNA and protein levels of IL-6 and TNFα after LPS pretreatment. BV-2 cells were pretreated with 1 μg/mL LPS for 24 h then 200-fold diluted essential oil chemotypes and standards were added to the pretreated BV-2 cells for 24 h. DMSO treatment was used as a control of the treated cells. Real-time PCR was performed with SYBR green protocol using IL-6 and TNFα specific primers. β-actin was used as housekeeping gene for the normalization and relative expression of controls was considered as 1. Proinflammatory cytokine secretions were determined using IL-6 or TNFα specific ELISA kits according to the manufacturer’s protocols. **a** Relative mRNA expressions of IL-6 and TNFα of the LPS pretreated cells. **b** IL-6 and TNFα ELISA measurements from the supernatant of the LPS pretreated BV-2 cells. The bars represent mean values and error bars represent standard deviation (SD) for three independent determinations (*n* = 3). Real-time PCR and ELISA measurements were carried out in triplicate in each independent experiments. Asterisks indicate *p* < 0.05 compared to the control. Crosses show *p* < 0.05 compared to the LPS treatment

At inflammation, BV-2 cells increase the production of the proinflammatory cytokines. Experiments were carried out to reveal whether thyme essential oil chemotypes and standards were able to ameliorate the effect of 24 h LPS pretreatment on the IL-6 and TNFα secretions of the BV-2 cells. Geraniol and thujanol chemotypes decreased the mRNA level of IL-6, while among the standards only geraniol was able to downregulate the IL-6 mRNA expression (Fig. 3a). At mRNA level, all three essential oil chemotypes decreased significantly the TNFα expression. Treatments with linalool and geraniol standards also resulted in the downregulation of TNFα mRNA levels (Fig. 3a). Linalool chemotype decreased IL-6 secretion of BV-2 cells after LPS pretreatment suggesting an anti-inflammatory effect of this essential oil (Fig. 3b). Geraniol and thujanol chemotypes could not decrease IL-6 production compared to the LPS treatment (Fig. 3b). In case of TNFα cytokine linalool, geraniol and thujanol chemotypes successfully reduced the TNFα secretion (Fig. 3b). Between the standards, linalool and geraniol significantly decreased both IL-6 and TNFα productions of the BV-2 cells, while thujanol was ineffective (Fig. 3b).

### Essential oil pretreatment alters the mRNA expression and secretion of IL-6 and TNFα of BV-2 cells exposed to LPS

**Fig. 4:**
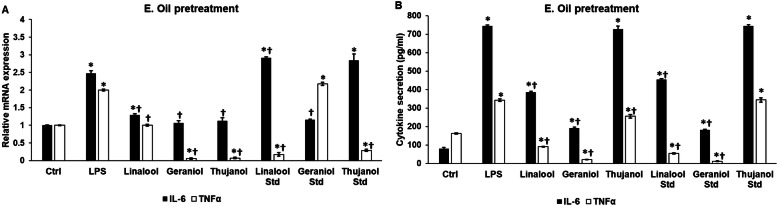
Effects of essential oil pretreatment on mRNA and protein levels of IL-6 and TNFα. BV-2 cells were pretreated with 200-fold diluted essential oils for 24 h then 1 μg/mL LPS was added to the pretreated BV-2 cells for 24 h. DMSO treatment was used as a control of the treated cells. Real-time PCR was performed with SYBR green protocol using IL-6 and TNFα specific primers. β-actin was used as housekeeping gene for the normalization and relative expression of controls was considered as 1. Proinflammatory cytokine secretions were determined using IL-6 or TNFα specific ELISA kits according to the manufacturer’s protocols. **a** Relative mRNA expressions of IL-6 and TNFα of the essential oil pretreated cells. **b** IL-6 and TNFα ELISA measurements from the supernatant of the essential oil pretreated BV-2 cells. The bars represent mean values and error bars represent standard deviation (SD) for three independent determinations (*n* = 3). Real-time PCR and ELISA measurements were carried out in triplicate in each independent experiments. Asterisks indicate *p* < 0.05 compared to the control. Crosses show *p* < 0.05 compared to the LPS treatment

Additional experiments were carried out to reveal whether a pretreatment of BV-2 cells with essential oils was capable to attenuate the inflammatory effect of LPS. Linalool, geraniol and thujanol chemotypes were able to decrease both the IL-6 and TNFα mRNA levels (Fig. 4a). Among essential oil standards, only geraniol was capable to downregulate IL-6 mRNA expression and interestingly geraniol was the only one that did not decrease the TNFα mRNA level. Linalool and thujanol standards were effective only against the elevated TNFα mRNA expression (Fig. 4a). At protein level, both chemotypes and standards of linalool and geraniol significantly decreased IL-6 secretion suggesting that might be a delay between mRNA expression and protein synthesis (Fig. 4b). The results of TNFα ELISA revealed that all essential oil chemotypes and standards decreased TNFα secretion except thujanol standard suggesting that pure thujanol essential oil acts through different pathways or it has distinct effect on TNFα synthesis (Fig. 4b).

### Effects of LPS and essential oil co-treatments on the mRNA expression and secretion of IL-6 and TNFα of BV-2 cells

**Fig. 5:**
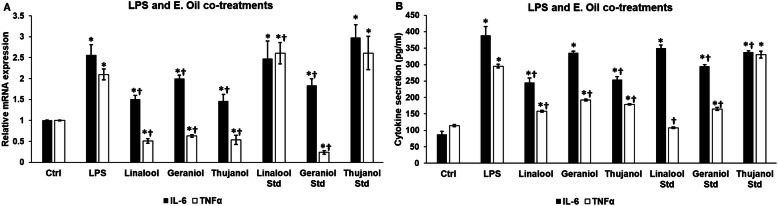
Effects of LPS and essential oil co-treatments on mRNA and protein levels of IL-6 and TNFα cytokines. BV-2 cells were treated with 1 μg/mL LPS and 200-fold diluted essential oils for 24 h. DMSO treatment was used as a control of the treated cells. Real-time PCR was performed with SYBR green protocol using IL-6 and TNFα specific primers. β-actin was used as housekeeping gene for the normalization and relative expression of controls was considered as 1. Proinflammatory cytokine secretions were determined using IL-6 or TNFα specific ELISA kits according to the manufacturer’s protocols. **a** Relative mRNA expressions of IL-6 and TNFα of the co-treated cells. **b** IL-6 and TNFα ELISA measurements from the supernatant of the co-treated BV-2 cells. The bars represent mean values and error bars represent standard deviation (SD) for three independent determinations (*n* = 3). Real-time PCR and ELISA measurements were carried out in triplicate in each independent experiments. Asterisks indicate *p* < 0.05 compared to the control. Crosses show *p* < 0.05 compared to the LPS treatment

BV-2 cells were treated parallel with LPS and essential oils to clarify whether the essential oil chemotypes and/or standards could modify the effect of LPS on the proinflammatory cytokine production of microglia. Essential oil chemotypes and geraniol standard were successful in decreasing the mRNA levels of IL-6 and TNFα cytokines (Fig. 5a). IL-6 mRNA level was also elevated by linalool and thujanol standards; moreover linalool significantly increased TNFα mRNA expression as well (Fig. 5a). All of the chemotypes, and among the standards only geraniol were capable to decrease both IL-6 and TNFα secretions (Fig. 5b). Linalool standard decreased only TNFα secretion while thujanol standard was effective against IL-6 production (Fig. 5b). Interestingly, thujanol standard increased TNFα secretion compared to the LPS treatment (Fig. 5b).

### Effects of essential oils on the NF-κB and phospho-C/EBPβ pathways regulating proinflammatory cytokine expression

**Fig. 6:**
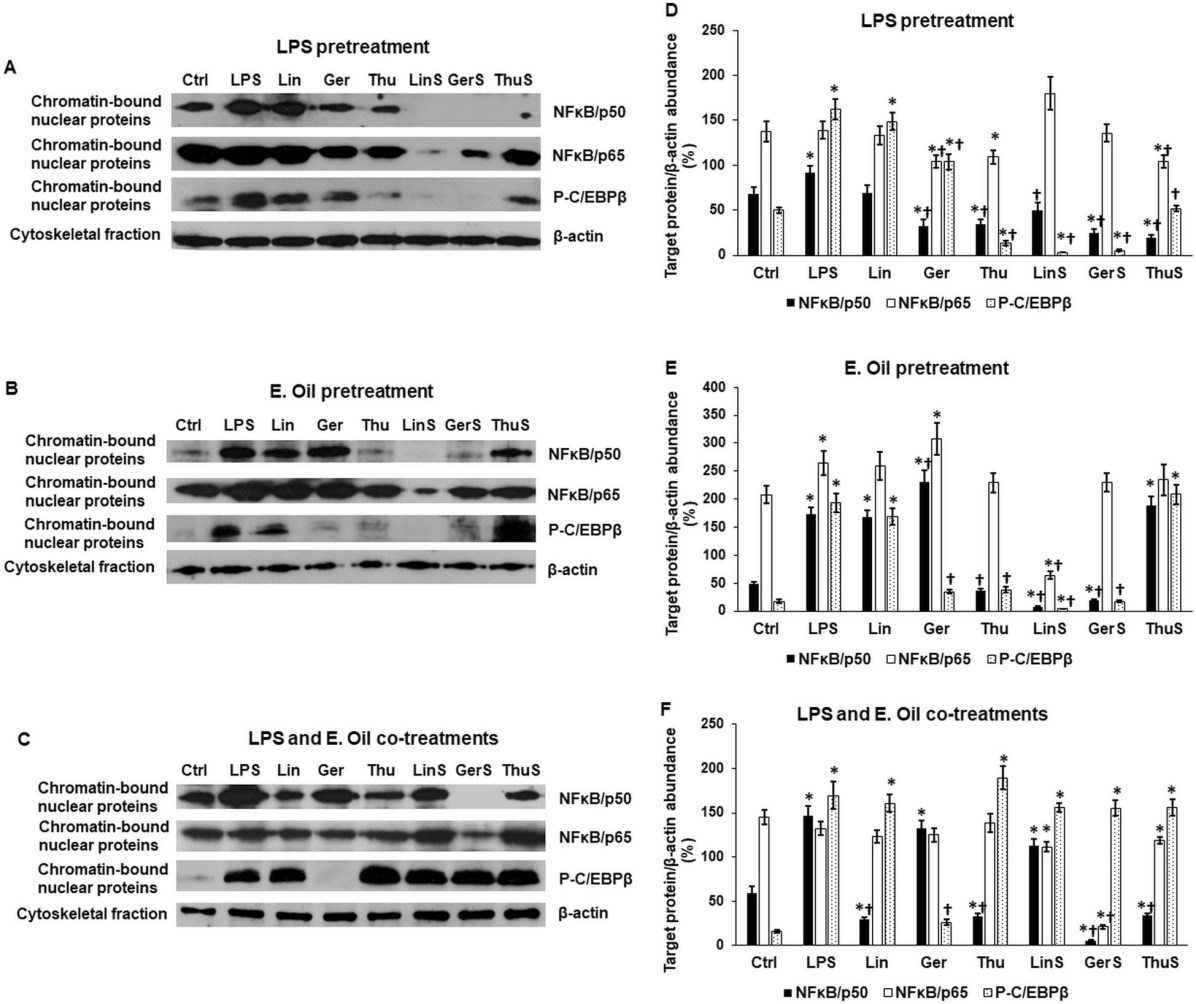
Western blot analyses of NFκB and phospho-C/EBPβ signalling pathways regulating proinflammatory cytokine production of BV-2 cells. BV-2 cells were fractionated immediately after collection and protein contents of the fractions were determined. The same amount of protein from each sample was loaded onto SDS-PAGE and transferred to nitrocellulose membranes then the membranes were probed with NFκB/p50, NFκB /p65 or P-C/EBPβ polyclonal rabbit antibodies according to the manufacturer’s protocol. β-actin was used as housekeeping control. **a** Protein levels of p50, p65 and P-C/EBPβ after LPS pretreatment. **b** Protein levels of p50, p65 and P-C/EBPβ after essential oil pretreatment. **c** Protein levels of p50, p65 and P-C/EBPβ after LPS and essential oil co-treatment. **d-f** Optical densities of the Western blot analyses of p50, p65 and P-C/EBPβ after the different treatments. The Western blots were analysed using ImageJ software, the optical density of the examined proteins was expressed as percentage of target protein/β-actin abundance. The bars represent mean values and error bars represent standard deviation (SD) for three independent experiments (*n* = 3). Asterisks indicate *p* < 0.05 compared to the control. Crosses show *p* < 0.05 compared to the LPS treatment. The protein samples from the same experiment were separated on different gels and only the target protein is visible on the blot used for the figure. Full-length blots are presented in Supplementary Fig. 1

LPS activates NF-κB pathway of microglia through toll-like receptor 4 (TLR4). After nuclear translocation, the NF-κB transcription factor activates the expression of proinflammatory genes (e.g. IL-6 and TNFα) by binding to their promoter regions [38, 39]. The effect of essential oil chemotypes and standards on the protein level of NF-κB using specific antibodies against p50 and p65 proteins were examined. In case of the LPS pretreatment following by essential oil treatments, the p50 level decreased by linalool and geraniol standards; meanwhile they did not change significantly the p65 protein level (Fig. 6a,d). Geraniol chemotype and thujanol standard decreased both p50 and p65 protein levels (Fig. 6a,d). Linalool chemotype did not affect the protein level of the examined NF-κB proteins.

Essential oil pretreatment partially changed the protein expression levels of NF-κB proteins regulated by LPS. Linalool standard decreased the examined protein levels (Fig. 6b,e). Thujanol chemotype and geraniol standard decreased p50 levels. Interestingly, geraniol chemotype altered only p50 level (Fig. 6b,e). Linalool chemotype acted the same was as in case of LPS pretreatment, it did not affect NF-κB protein expressions (Fig. 6b,e).

LPS and essential oil co-treatments revealed completely different results compared to the former experiments. Geraniol standard was the most effective against NF-κB proteins; it decreased the levels of all of the examined proteins. However, geraniol chemotype did not change the levels of any of these proteins (Fig. 6c,f). Geraniol standard decreased both NF-κB protein levels, but geraniol chemotype did not. Thujanol chemotype and standard only decreased p50 protein level. Linalool chemotype successfully decreased p50 protein level, while linalool standard did not exert any changes on NF-κB proteins (Fig. 6c,f).

Based on these Western blot results it seems that geraniol standard has the most powerful effect on the LPS-stimulated inflammatory response. Geraniol chemotype and linalool and thujanol standards were also successful in decreasing the effect of LPS treatment on NF-κB pathway. Thujanol chemotype and standard and linalool standard could prevent NFκB activation at LPS treatment.

The alterations of the NF-κB signalling pathway could not explain completely the changes in the TNFα expression; therefore, the level of the P-C/EBPβ transcription factor, which has been described as a possible TNFα regulator in myelomonocytic cell lines [37] was also examined. All of the essential oil standards and among the chemotypes geraniol and thujanol could attenuate the effect of LPS pretreatment on P-C/EBPβ transcription factor (Fig. 6a,d). In case of essential oil pretreatment the aforementioned chemotypes and standards successfully decreased P-C/EBPβ protein level with the only exception thujanol standard (Fig. 6b,e). Interestingly, the co-treatment of BV-2 cells with LPS and essential oils did not affect the level of P-C/EBPβ transcription factor, only geraniol chemotype decreased significantly the P-C/EBPβ protein level (Fig. 6c,f). Geraniol chemotype was the only essential oil, which was able to decrease P-C/EBPβ level in each experiment. Moreover, this chemotype was also successful in preventing the effect of LPS treatment on NFκB pathway. Thujanol chemotype and linalool, geraniol standards were also capable to prevent the LPS-induced C/EBPβ activation. The only essential oil that was ineffective in C/EBPβ activation in all three types of experiments was linalool chemotype. Moreover, linalool chemotype produced no effect on the NFκB activation, too.

## Discussion

Large number of medicinal herbs and their extracts are used for treatments of various diseases. One of the most frequently examined plant is thyme (*Thymus vulgaris* L.). Different chemotypes of the essential oils of this plant have been tested in the past and proved their antibacterial and antifungal activities, though at various effectiveness against the certain microbes [21, 40].

It is clear that there are fundamental differences among essential oils and their chemotypes not only in their therapeutic but also in their basic effects on cultured cells [41], which was proved by the viability assays carried out in time and concentration dependence. In addition plant extracts behave distinctly depending on the target cell: in a numerous experiments they were used not on cultured cells but against microbes directly [42].

Nowadays it has been accepted that inflammation is playing a determining role in the development and seriousness of neurodegenerative diseases, like Alzheimer’s disease, Parkinson’s disease or multiple sclerosis [43]. In the central nervous system astrocytes and microglia are responsible for mediating the immunoresponse against inflammatory agents [43]. The essential oils are lipophilic and organic molecules, which are able to transfer across the epithelium in nasal mucosa. Upon passing through the epithelium they move into systemic circulation and cross the blood-brain barrier [16], although the components of the essential oils show different permeability via the blood-brain barrier [17]. Cheng et al. described that linalool can pass through the blood-brain barrier in mice [44] and can reverse both neuropathological and behavioural impairments [45]. Geraniol was successful in decreasing the impairments of motor behaviour in mouse model of Parkinson’s disease [46]. Based on the neuroprotective and anti-aging effects of essential oils they can be used as complementary therapy in age related neurodegenerative diseases such as Parkinson’s disease, Alzheimer’s disease, Huntington’s disease and amyotrophic lateral sclerosis [47].

We have been investigating the potential protective effect of *Thymus vulgaris* essential oil chemotypes and their main compounds in an in vitro microglial cell culture system. As a model LPS was used to imitate bacterial infection in cell cultures and tested the effects of chemotypes of thyme essential oil [48–50]. Relatively little is known about the interactions of these essential oils and the cells of the nervous system*.* By Elmann et al. the effects of geranium oil was examined in primary rat microglia cell culture as the protective agent against inflammation mediated by LPS administration [51]. Essential oil was added at the same time with LPS to the cultured cells. According to the authors, NO release and the expression of inducible NO synthase and cyclooxygenase 2 were reduced by the geranium oil treatment. These experiments are modelling neurodegenerative diseases which are related to neuroinflammation.

The post ischemic processes in the brain involve a large number of components. Among them the leaders are the proinflammatory cytokines, which are playing a basic role in the worsening of the damage of the blood brain barrier as well as the activation of microglial cells. The protective effect of linalool was examined in rats after a period of cerebral arterial occlusion [52]. Also primary glial cells suffered less damage after glutamate challenge when treated with linalool. It is interesting that linalool was administered intranasal proving that these plant compounds can reach the cells of the central nervous system via the blood brain barrier by inhalation.

BV-2 rodent microglia are widely accepted models for examining those agents, which may be involved in induction of neurodegeneration by microglia activation. LPS is a component of the cell wall of Gram- negative bacteria and may be used in microglia cell culture to mimic inflammation and for testing potential anti-inflammatory molecules [53]. The advantages of model cell culture experiments are the possibility of large number of variations in controlled circumstances. The temperature, composition of the cell culture medium are the same, while the timing and the concentrations of the different treatments are variable. Also it is possible to carry out the treatments in different order or at the same time. With these setups it was possible to imitate the preventive effect or the therapeutic effects of the different pharmaceutical molecules. In addition, there is a possibility to compare essential oils, chemotypes from the market, or produced in-house.

In our work definite alterations were revealed in the effects of essential oil chemotypes and their main compounds at the different experimental setups. These cellular changes were followed at mRNA and at protein levels in LPS treated cells and in cells without LPS challenge. In general the reduction in proinflammatory IL-6 and TNFα syntheses and secretions were seen at the presence of treatments.

A couple of comparisons were carried out in our experiments: the effects of three different chemotypes and standards on the survival of BV-2 cells as well as on the synthesis and secretion of IL-6 and TNFα. The latter changes were followed together with LPS treatment, in three versions: pretreatment with LPS, pretreatment with essential oils, or co-treatment with the two types of substances. We examined the NF-κB signalling pathway and the TNFα regulation activity in each of the three treatment versions mentioned above.

Essential oils by themselves had effect on IL-6 and TNFα mRNA syntheses and secretions. It was revealed several times that the mRNA synthesis and cytokine release are not always changing parallel. The protein synthesis and posttranslational modifications may have different regulatory signals than transcription or it is a possibility that the former processes need more time than the mRNA synthesis. The two proinflammatory cytokines show the same secretion pattern, with chemotypes e.g. linalool and geraniol exerting stronger effects. This phenomenon can be observed frequently, suggesting that a pure, single component can have limitations, a “mixture” in a plant extract is having more active compounds that may cooperate against inflammation. Elman et al. found that a single component of essential oil did not exert protective effect in neuroinflammation examined in primary microglia cells [51].

Considering the changes of IL-6 and TNFα secretions depending on the relation of LPS and essential oil treatments in time, the best reduction of inflammatory cytokines could be reached by the pretreatment with the essential oils. In these experiments, there were a few surprises. In every setup (LPS pretreatment, essential oil pretreatment, co-treatment of LPS and chemotypes), the reduction of TNFα were grater, than that of the IL-6 at both mRNA and protein levels. In addition, occasionally standards had better effects, than chemotypes, but mainly at mRNA expression. At the essential oil pretreatment experiment the effect of linalool and geraniol was outstanding, proving at least two facts. Using these plant materials as prophylaxis against inflammation (or at least LPS effect) showed the best result. Also according to the research of other scientific groups these two substances in both standard and chemotype forms have specific effects on microglia/macrophages in neuronal injury, hypoxia and degeneration.

To explain the reason of alterations in proinflammatory cytokine production of BV-2 microglia, the transcription factor components of NF-κB, namely the level of chromatin-bound, active p50 and p65 were examined. When they act in heterodimer form [54, 55], they activate the transcription of IL-6 and TNFα. Interestingly, standards caused large reduction of chromatin-bound proteins, especially linalool, both in the case of LPS pretreatment as well as in essential oil administration before LPS. Further investigating activations of cell signalling pathways, the level of chromatin-bound, phosphorylated C/EBPβ was determined Large differences could be seen in the levels of P-C/EBPβ in the effects of standards and chemotypes and co-treatments and pretreatments. Best effects could be observed in geraniol and thujanol chemotypes and linalool and geraniol standards, the latter ones in pretreatment only.

The presence of the additional components in the essential oil chemotypes revealed by GC-MS method may contribute to the effect of the main compounds (Table 3). It was proven that p-cymene, found in both thujanol and linalool chemotypes in 2.1 and 4.2%, possessed anti-inflammatory effects in mice and decreased leukocyte migration [56]. γ-Terpinene found in the thujanol chemotype (2.9%) as well as α-terpineol (4.3%) were previously described as anti-inflammatory molecules, the latter one was able to decrease IL-6 mRNA level [57, 58]. The terpinen-4-ol can suppress the production of inflammatory mediators in macrophages [59] and may interact with the main components of thujanol (11.9%) and linalool (8.3%) thyme essential oil chemotypes. Linalyl acetate found in thujanol chemotype (2.9%) has been proven to provide anti-inflammatory effect on natural killer cell in a dose dependent manner [60]. Nerol was also found in both geraniol (1.9%) and thujanol (4.2%) chemotypes, which was proven to decrease IL-13 and TNFα pro-inflammatory cytokines [61]. The anti-inflammatory effect of geraniol chemotype may be supported by the presence of geranyl acetate (18.6%), β-caryophyllene (5.7%), geranyl propionate (2.2%) and elemol (2.2%) [62–65]. The differences in the composition of the examined thyme essential oil chemotypes may contribute to their distinct effects on the regulation of IL-6 and TNFα pro-inflammatory cytokine syntheses in BV-2 microglia.

Based on our observations it can be concluded that geraniol (both chemotype and standard) has an outstanding effect on decreasing pro-inflammatory cytokine secretion. Moreover, the presence of additional components in the chemotypes may alter the effect of the main compounds since the chemotypes have better effect alone at the treatments, but in the presence of LPS (LPS pretreatment and LPS and essential oils together) they can achieve weaker inhibitory effect on the production of pro-inflammatory cytokines.

In summary, we may declare that BV-2 cells are good models to examine the neuroprotective effects of essential oil of thyme (*Thymus vulgaris* L.). These protective effects are caused by not the same components, which are responsible for the antibacterial effect of thyme. In many aspects essential oil chemotypes are more effective than standards, but standards could be seen to have large inhibitory effects on certain cell signalling components related to the activation of proinflammatory cytokines. This proves that the final change in the secreted levels of IL-6 and TNFα could not be explained merely by one transcription factor activity. There is also a possibility that the change in the activation of transcription factors is occurring in a different time frame than the examined period in our experiments.

## Conclusions

*Thymus vulgaris* essential oil and its linalool and geraniol chemotypes are good candidates to use in prevention of neuroinflammation and related neurodegeneration, but the exact ratio of the components has to be selected carefully. To map the signalling pathways of the compounds further experiments need to be carried out.

## Supplementary Information

**Additional file 1.**

## Data Availability

All data generated or analysed during this study are included in this published article.

## Abbreviations

C/EBPβ: CCAAT-enhancer binding protein β
CNS: Central nervous system
DMSO: Dimethyl sulfoxide
FBS: Fetal bovine serum
GC MS: Gas-chromatography-mass spectrometry
HRP: Horseradish peroxidase
IL-6: Interleukin-6
LPS: Lipopolysaccharide
NFκB: Nuclear factor kappa -light-chain-enhancer of activated B cells
NO: Nitric oxide
TBST: Tris buffer saline with Tween
TNFα: Tumor necrosis factor α
