# Supplementary material for: Three chemotypes of thyme (Thymus vulgaris L.) essential oil and their main compounds affect differently the IL-6 and TNFα cytokine secretions of BV-2 microglia by modulating the NF-κB and C/EBPβ signalling pathways
Source: BMC Complement Med Ther. 2021 May 22;21:148. doi: 10.1186/s12906-021-03319-w (PMC8140451; doi:10.1186/s12906-021-03319-w)

Three chemotypes of thyme (*Thymus vulgaris* L.) essential oil and their main compounds affect differently the IL-6 and TNFα cytokine secretions of BV-2 microglia by modulating the NF-κB and C/EBPβ signalling pathways

Györgyi Horváth [1], Adrienn Horváth [1], Gréta Reichert [1], Andrea Böszörményi [2], Katalin Sipos [3] and Edina Pandur [3,*]

[1] Department of Pharmacognosy, Faculty of Pharmacy, University of Pécs, H-7624, Rókus u. 2., Pécs, Hungary; horvath.gyorgyi@gytk.pte.hu, horvath.adrienn2@pte.hu, reichertgreta@icloud.com

[2] Institute of Pharmacognosy, Faculty of Pharmacy, Semmelweis University, H-1085, Üllői út 26. Budapest, Hungary; boszormenyi.andrea@pharma.semmelweis-univ.hu

[3] Department of Pharmaceutical Biology, Faculty of Pharmacy, University of Pécs, H-7624, Rókus u.2., Pécs, Hungary; katalin.sipos@aok.pte.hu, edina.pandur@aok.pte.hu

* Correspondence: edina.pandur@aok.pte.hu

Supplementary Figure 1. Original Western blots. **(A)**WBs showing p50, p65, P-C/EBPβ and β-actin protein levels after LPS pretreatment of BV-2 cells. **(B)** WBs showing protein levels of p50, p65 and P-C/EBPβ after essential oil pretreatment. **(C)** WBs showing protein levels of p50, p65 and P-C/EBPβ after LPS and essential oil co-treatment. The Western blots were performed using manual developing method. The development was made on X-Ray films, then the films were scanned using automatic grey scale mode and 600 dpi.

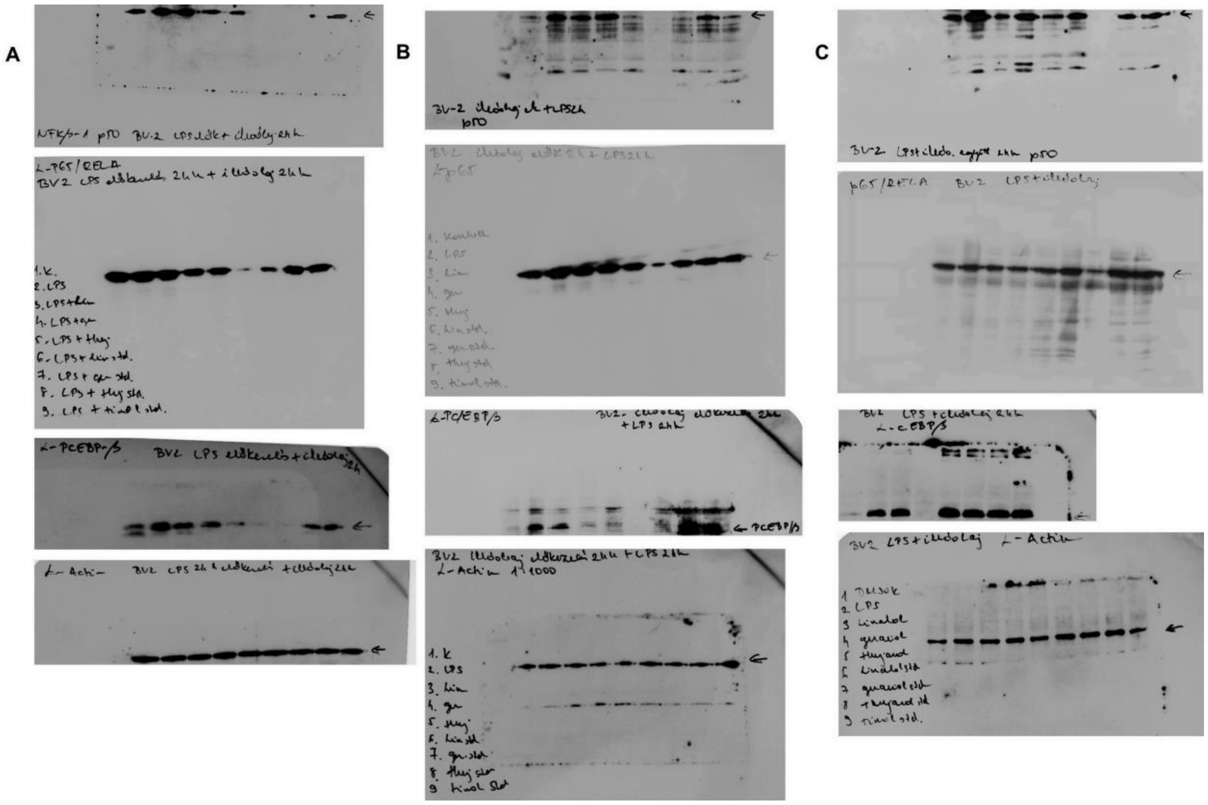

Supplement: Supplementary file 1 — Additional file 1. [file 12906_2021_3319_MOESM1_ESM.pdf]
